# The relationship between trauma, shame, and guilt: findings from a community-based study of refugee minors in Germany

**DOI:** 10.3402/ejpt.v6.25863

**Published:** 2015-06-22

**Authors:** Sabrina J. Stotz, Thomas Elbert, Veronika Müller, Maggie Schauer

**Affiliations:** Department of Psychology, University of Konstanz, Konstanz, Germany

**Keywords:** Shame, guilt, trauma exposure, posttraumatic stress disorder, refugee minors

## Abstract

**Background:**

The relationships between traumatic stress and self-conscious emotions, such as shame and guilt, remain to be fully explored, especially in refugees, who frequently are exposed to a multitude of stressors.

**Objective:**

The aim of the present study was to investigate shame and guilt in refugee minors and to assess to what extent a greater cumulative exposure to traumatic stressors would result not only in more severe posttraumatic stress disorder (PTSD) symptoms but also in higher levels of shame and guilt.

**Methods:**

Thirty-two male refugee minors, who were all below the age of 18 when they sought asylum in Germany, agreed to participate. At the time of the assessment, the age ranged from 11 to 20 years. Eighteen refugees had arrived without relatives in their host country (“unaccompanied minors”). In structured diagnostic interviews, a PTSD diagnosis was established using the UCLA PTSD Index. Posttraumatic guilt was assessed by means of the Trauma-related Guilt Inventory, and the Shame Variability Questionnaire was used to record the intensity, duration, and frequency of shame episodes.

**Results:**

Feelings of guilt and shame as well as trauma symptoms were all associated with the number of traumatic event types subjects had experienced. Posttraumatic guilt and shame were both correlated with PTSD symptom severity.

**Conclusions:**

The findings indicate that cumulative stress such as exposure to multiple traumatic events poses a risk factor for the mental health including greater suffering and functional impairment due to shame and guilt.

Ample research has demonstrated that the cumulative severity of exposure to psychological stressors renders a person more vulnerable to developing posttraumatic stress disorder (PTSD) and indicates a strong relationship between the magnitude of traumatic events and the severity of PTSD symptoms (Neuner et al., 2004; Schaal & Elbert, 2006; Schauer et al., 2003). Victims and survivors of war (Leskela, Dieperink, & Thuras, 2002), violent crime (Semb, Stömsten, & Sundbom, 2011), and abuse (Andrews, Brewin, Rose, & Kirk, 2000) suffer from significant feelings of shame and guilt closely related to the traumatic experiences. Both these self-conscious emotions are common in traumatized refugees. This may appear counterintuitive at first sight, because refugees have, in many cases, been victims of harsh conditions beyond their control, such as war, torture, lack of food, or natural disasters. However, subjective feelings of shame and guilt may be associated with an objectively unforeseeable and uncontrollable event.

In accordance with Kubany and Watson (2003), guilt is defined as “an unpleasant feeling with accompanying beliefs that one should have thought, felt, or acted differently” (p. 53). It is considered to be multidimensional as well as a function of its components: distress, and four interrelated beliefs about one's role in the traumatic event comprising guilt cognitions. Furthermore, situational factors, such as direct involvement or harm caused to a close relationship partner, may contribute to guilt and account for the fact that individuals exposed to similar or even the same event experience disparate levels of guilt (Kubany & Watson, 2003). Although both guilt (“I should not have done **this**.”) and shame (“**I** should not have done this.”) are self-conscious emotions elicited by the same kinds of negative events (Tangney, Burggraf, & Wagner, 1995), it is important to differentiate guilt from shame. In this context, shame is defined as an unpleasant feeling involving a global negative self-evaluation, taking the perspective of the others. Thus, the essential distinction is the involvement of the anticipated social devaluation of the self in shame, whereas guilt involves a deprecation of specific behaviors, actions, or thoughts. Substantial evidence suggests that intense feelings of guilt in response to action taken or failure to act immediately prior to or during the event are highly correlated with the severity of PTSD symptoms (Kinzie, Sack, Angell, Manson, & Rath, 1986). In addition, long-lasting shame episodes have been found to be a significant predictor for PTSD symptoms (Andrews et al., 2000; Tangney, Wagner, & Gramzow, 1992). Thus, both shame and guilt may have profound implications for research and trauma therapy (Kubany & Watson, 2003).

Research data on mental health of refugee minors have remained limited. The aim of the present study was to determine the relationships between shame and guilt with the extent of traumatic events experienced in relation to psychological distress. Refugee minors, children under 18 years of age, are a particular vulnerable group that is not only affected by direct exposure to traumatic events but also by secondary adverse effects, such as the loss of loved ones, the absence of one or both parents, lack of family support, family conflict, and violence or ruptures in daily routines (Fazel & Stein, 2002; Geltman et al., 2005; Lustig et al., 2004). In this study, group comparisons were conducted of unaccompanied minor refugees (UMR) who have been separated from both parents and accompanied minor refugees (AMR) who arrived in Germany with a guardian. This allowed the exploration of self-conscious emotions and psychopathology, taking account of the distinct variation in social contexts between both groups.

## Methods

### Participants

Initial contact with the refugees was only possible through authorities, community accommodations, and other social facilities. The involvement of responsible social workers facilitated the recruitment as they introduced the goal and context of the investigation to the adolescents before they suggested contacting the investigators. Because of confidentiality already at this stage, it is not possible to present figures about the number that declined to participate in the study. Eighteen unaccompanied and fourteen accompanied adolescent refugees from various parts of southern Germany agreed to participate in the study. All of them were males aged between 11 and 20 years at the time of evaluation. However, all participants had entered Germany as minors. In the following, the authors refer to them as minors to highlight this fact. On average, unaccompanied minors (*M=*17.4, *SD=*0.98) were about 2 years older than their accompanied peers (*M=*15.6, *SD=*2.21). As main reasons for their escape, the adolescents stated war (32%) and prosecution by extremist groups (27%) in their home country, family conflicts (27%), and the hope for a better life (11%) in Germany, as well as other reasons (3%). Home countries were diverse and included Afghanistan, Iran, Iraq, as well as African (e.g., Gambia, Nigeria, or Sierra Leone), and European countries (e.g., Kosovo, Serbia, or Turkey). On average, participants had entered Germany 39.86 months (*SD=*49.77; range 1.5–197) before their interviews.

### Procedure

The present study was conducted from September 2011 to March 2012. Half of the interviews took place at the Center for Traumatic Stress Studies, University of Konstanz, Germany. The other half of the interviews was conducted at the place of residence of the refugee. Interviews were conducted by trained psychologists of the center in German or English. About one-third of the interviews involved trained interpreters and were held in the minors’ native language.

### Clinical assessment

In structured diagnostic interviews, the number of traumatic events was assessed with a checklist and all participants were screened for PTSD using the UCLA PTSD Index (Steinberg, Brymer, Decker, & Pynoos, 2004). Posttraumatic guilt was assessed by means of the Trauma-related Guilt Inventory (TRGI; Kubany, 2004; Kubany et al., 1996). The TRGI is an event-focused self-report measure of trauma-related guilt. The instrument consists of three subscales: “global guilt” (e.g., “I experience intense guilt related to what happened”), “distress” (e.g., “What happened causes a lot of pain and suffering”), and “guilt cognitions” (e.g., “I was responsible for causing what happened”). Participants were asked to identify the most distressing event (or series of events) and to rate their responses to this particular event on a 5-point Likert scale, ranging from 0 (Not at all true or Never true) to 4 (Extremely true or Always true). Whereas the “distress” scale measures the affective response to the event(s), the “total guilt cognitions” score represents the cognitive components of trauma-related guilt (Kubany et al., 1996). The “global guilt” scale assesses the overall guilt and can be best understood in terms of a combination of distress and the guilt-related beliefs that exacerbate the distress. Scores were transformed into T-scores, with scores ≥60T are considered to be clinically significant. The TRGI allowed for the assessment of trauma-related guilt in response to a specific event or series of events, which in turn could be further examined using the UCLA PTSD Index. Because scenario-based inventories such as the commonly used Test of Self-Conscious Affect (Tangney, Wagner, Gavlas, & Gramzow, 1991) were primarily designed for application in the western world and do not match the daily life of the refugee minors, we recorded the intensity, duration, and frequency of shame episodes experienced in the past 4 months using the Shame Variability Questionnaire (SVQ; Brown et al., unpublished). Participants were asked to remember a specific time over the last 4 months when they felt the worst about themselves and to rate the intensity of shame-related experiences (e.g., “I hated myself,” “I felt that I was a bad person,” and “I felt that I deserved to be punished”) on a 5-point Likert scale from 1 (Not at all, I did not feel this way) to 5 (Completely, I felt this way very strongly). In addition, the duration of feelings of shame during the worst episode (6-point Likert scale) as well as the frequency of episodes of similar severity (5-point Likert scale) were assessed. Pathological feelings of shame are marked by high intensity, long duration, and high frequency. Therefore, the SVQ analysis variable of clinically significant shame responses is a combination of intensity*duration*frequency. Results from clinical and non-clinical samples indicate that the SVQ has high internal consistency, test–retest reliability, and criterion validity (Brown et al., unpublished).

Moreover, the mental health and family background, including parents’ rearing behavior and family violence, were also assessed but are not reported here.

## Results

### Trauma exposure

Refugee minors were exposed to a wide range of traumatic stressors. The mean number of separate traumatic events experienced by the accompanied minors was three. However, on average, UMR reported that they had experienced seven different traumatic event types. These mean scores differ significantly (*U=*30.5, *p<*0.001, *r=*0.651). Most distressing were war and armed conflict (16%), violent death, or injury of a beloved one (14%), imprisonment and torture (11%), witnessing or experiencing injury by a weapon or a gun (8%), family violence (5%), recruitment as a child soldier (5%), sexual abuse (3%), and experiencing a dramatic accident (3%).

### Level of psychological distress

Overall, nine (28.1%) of the refugee minors fulfilled the DSM-IV-TR criteria for a PTSD diagnosis. The UMR showed higher symptom scores (*M=*22.83; *SD=*18.44) than AMR (*M=*3.1; *SD=*4.8) and more often received a diagnosis of PTSD (Table 1). Mann–Whitney tests revealed significant group differences for the PTSD sum score (*U=*46, *p=*0.002, *r=*0.547) and the subscales, intrusions (*U=*50, *p=*0.003, *r=*0.525), avoidance (*U=*51, *p=*0.003, *r=*0.522), and hyperarousal (*U=*35.5, *p<*0.001, *r=*0.630).

**Table 1:** Descriptive data and statistical differences between both groups

	Accompanied		Unaccompanied		
		
	*N*	Mean (*SD*)	*N*	Mean (*SD*)	Statistical difference
Age (years)	14	15.64 (2.21)	18	17.4 (0.98)	*U=*70.5, *p=*0.024, *r=*0.40
Percentage with PTSD	14	0% (*n=*0)	18	50% (*n=*9)	χ^2^ *=*9.7, *p=*0.002, ϕ*=*0.552
PTSD symptom score	14	3.1 (4.80)	18	22.83 (18.44)	*U=*46, *p=*0.002, *r=*0.547
Intrusions	14	1.0 (1.52)	18	6.78 (5.95)	*U=*50, *p=*0.003, *r=*0.525
Avoidance	14	1.29 (2.30)	18	8.78 (7.82)	*U=*51, *p=*0.003, *r=*0.522
Hyperarousal	14	0.86 (1.61)	18	7.28 (5.61)	*U=*35.5, *p<*0.001, *r=*0.630
Global guilt	11	43.64 (7.98)	16	48.56 (14.39)	ns
Total guilt cognitions	11	43.55 (10.58)	16	50.69 (10.61)	*U=*47.5, *p=*0.045, *r=*0.354
Distress	11	43.64 (8.39)	16	61.12 (10.65)	*U=*15.5, *p<*0.001, *r=*0.634
Intensity*duration*frequency of shame	14	9.89 (7.24)	18	16.76 (10.16)	*U=*65, *p=*0.020, *r=*0.41
Intensity of shame	14	2.37 (0.67)	18	2.63 (0.94)	ns
Intensity*duration of shame	14	5.91 (2.67)	18	7.31 (3.24)	ns

PTSD, posttraumatic stress disorder; ns, not significant; *SD*, standard deviation.

### Levels of shame and guilt

In the present study, the prevalence of trauma-related guilt is 18.5% for global guilt, 25.9% for the affective component, and 22.2% for the cognitive component of guilt. UMR demonstrated a mean score of 48.56 (*SD=*14.39) and, therefore, experienced higher levels of global guilt than their accompanied peers (*M=*43.64, *SD=*7.98). Mean scores of both groups also differed significantly for the affective (*U=*15.5, *p<*0.001, *r=*0.634) and cognitive (*U=*47.5, *p=*0.045, *r=*0.354) components of guilt. However, no significant group differences were identified for global guilt (Table 1).

A descriptive analysis of data assessed by means of the SVQ revealed an average intensity*duration*frequency total of 13.75 (*SD=*9.51, range 2.29–42.43) in the refugee sample. Thereby, pathological shame is characterized by shame episodes of high intensity, duration, and frequency. Mean SVQ subscale scores were 2.52 for the intensity (*SD=*0.83, range 1.21–4.71) and 6.7 for intensity*duration of shame episodes (*SD=*3.04, range 2.29–14.14). In accordance with previous results, unaccompanied minors showed shame episodes of higher intensity*duration*frequency than their accompanied peers (*U=*65, *p=*0.020, *r=*0.41). However, no significant group differences were identified for the intensity and intensity*duration scores (Table 1). In addition, all components of guilt were found to be positively related to shame. A stepwise regression displayed the affective component (β=0.30, *t=*2.42, *p=*0.024, *f* ^2^=1.04) and global guilt (β=0.32, *t=*2.43, *p=*0.023, *f* ^2^=1.04) as significant predictors of pathological feelings of shame. The final model explained 51% of the variance in the outcome variable [*R* ^2^=0.51, *F*(2,26)=12.28, *p<*0.001].

### Relationship between self-conscious emotions and PTSD symptoms

In the present study, both shame and guilt were found to be strongly related to the severity of PTSD symptoms experienced by the refugee minors. PTSD sum scores showed a significant correlation with global guilt (*r=*0.52, *p=*0.005) as well as its affective (*r=*0.76, *p<*0.001) and cognitive (*r=*0.46, *p=*0.016) components. In addition, significant associations between the subscales, intrusion, avoidance, and hyperarousal with global guilt and its components were recorded. Furthermore, the data show significant correlations between the PTSD sum score and the SVQ intensity*duration*frequency score (*r=*0.67, *p<*0.001). All subscales were also found to be significantly correlated.

Mann–Whitney tests were used to test whether refugee minors who had reached the UCLA PTSD Index cut-off score ≥24, and therefore received a PTSD diagnosis, would also experience higher levels of shame and guilt than their peers without a diagnosis. Mean scores of the affective component of guilt differed significantly (*U=*7, *p<*0.001, *r=*0.71), but not for the cognitive component and global guilt. Moreover, significant group differences could be found for the intensity*duration*frequency total (*U=*23, *p=*0.001, *r=*0.596). A stepwise regression displayed the PTSD symptom severity as a significant predictor of pathological feelings of shame (β=0.37, *t=*4.97, *p<*0.001, *f* ^2^=0.82). The final model explained 45% of the variance in the outcome variable [*R* ^2^=0.451, *F*(1,31)=24.65, *p<*0.001]. Thus, the current results show that individuals who reported higher levels of psychological distress, and therefore received a diagnosis, also experienced significantly higher levels of guilt-related distress, as well as pathological feelings of shame.

### Relationship between self-conscious emotions and cumulative psychological trauma

**Fig. 1:**
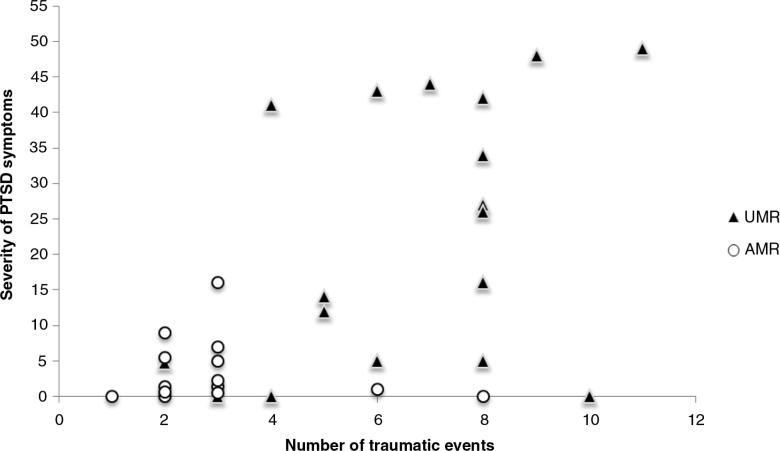
Scatterplot of the total number of traumatic events and the severity of PTSD symptoms experienced by unaccompanied (UMR) and accompanied (AMR) minor refugees.

To examine the relationship between PTSD symptoms and the experienced number of traumatic events, the UCLA PTSD Index sum score and its subscales, intrusion, avoidance, and hyperarousal were correlated with the number of events reported by the adolescents. Findings indicate that the number of traumatic events in life is significantly correlated with the severity of intrusions (*r=*0.57, *p<*0.001), avoidance (*r=*0.54, *p=*0.001), and hyperarousal (*r=*0.58, *p<*0.001). The sum score of the UCLA PTSD Index displays a highly significant association with the reported number of traumatic events (*r=*0.58, *p<*0.001; Fig. 1).

**Fig. 2:**
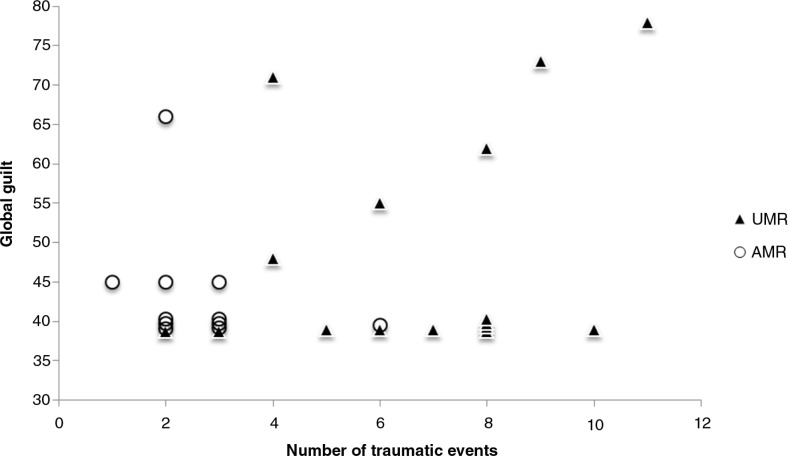
Scatterplot of the total number of traumatic events and global guilt experienced by unaccompanied (UMR) and accompanied (AMR) minor refugees.

To test the hypothesis that a higher number of traumatic events are associated with a higher level of guilt, Pearson correlation coefficients were computed. According to expectations, the number of trauma exposures showed a positive correlation with the guilt-related distress experienced (*r=*0.51, *p=*0.007). However, no significant associations of trauma exposure and total guilt cognitions (*r=*0.244, *p=*0.220) or the reported level of global guilt (*r=*0.273, *p=*0.168) could be identified (Fig. 2). To ensure sufficient power to examine the proposed associations of trauma exposure with total guilt cognitions and global guilt, a power analysis was conducted using G*Power 3 (Faul, Erdfelder, Lang, & Buchner, 2007). A *post hoc* power analysis with the given correlations, an alpha level of 0.05, and a total sample size of 27 indicated achieved power levels of 0.28 and 0.23. As further analysis revealed, the present study would have sufficient power (i.e., at least 0.80) to detect medium (*r=*0.30) and large effects (*r=*0.50) for all three TRGI subscales with a minimum sample size of 129.

**Fig. 3:**
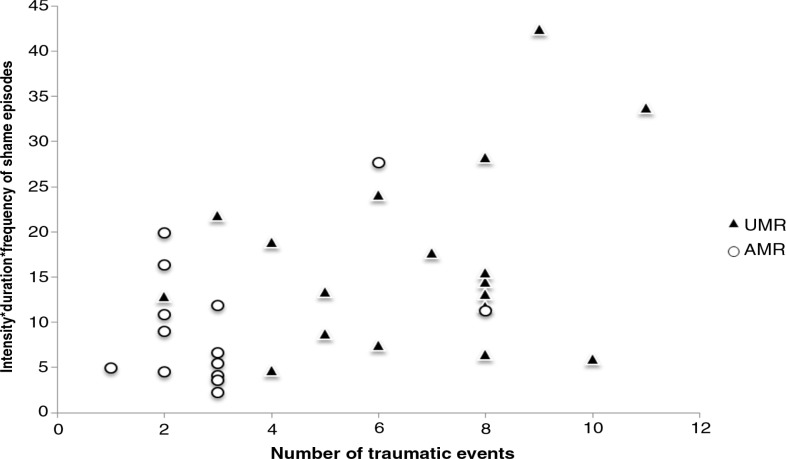
Scatterplot of the total number of traumatic events and intensity*duration*frequency of shame episodes experienced by unaccompanied (UMR) and accompanied (AMR) minor refugees.

As shown in Fig. 3, the intensity*duration*frequency of shame episodes experienced by refugee minors was strongly related to the reported number of traumatic events (*r=*0.46, *p=*0.008). Similarly, a significant relationship between the number of traumatic events experienced and intensity*duration of shame episodes (*r=*0.403, *p=*0.022) could be found. Furthermore, a stepwise regression displayed the number of traumatic event types as a significant predictor of pathological feelings of shame (β=1.55, *t=*2.84, *p=*0.008, *f* ^2^=0.27). The final model explained 21% of the variance in the outcome variable [*R* ^2^=0.21, *F*(1,31)=8.06, *p=*0.008]. In conclusion, although the proposed cumulative effect of multiple trauma exposure, known as the “building block” effect (Neuner et al., 2004; Schauer et al., 2003), could not be identified for the overall level of guilt, findings corroborate our assumptions indicating that the exposure to multiple traumatic events is strongly associated with higher intensity*duration*frequency of shame episodes.

## Discussion

The present study examined the relationship between trauma exposure and self-conscious emotions, and their cumulative effects on mental health of refugee minors residing in southern Germany.

In accordance with previous European and US studies (e.g., Bean, Derluyn, Eurelings-Bontekoe, Broekaert, & Spinhoven, 2007; Derluyn, Broekaert, & Schuyten, 2008; Geltman et al., 2005; Hodes, Jagdev, Chandra, & Cunniff, 2008), UMR reported a higher number of traumatic events and more often received a PTSD diagnosis than their accompanied peers.

The probability of detecting a relationship between trauma exposure and the PTSD symptoms depends on the range of frequencies and variance of trauma exposure. Both are substantial in this sample. The number of traumatic events was found to be significantly associated with the severity of PTSD symptoms experienced by the adolescents. Hence, in agreement with previous research (Bean et al., 2007; Derluyn, Mels, & Broekaert, 2009; Ellis, MacDonald, Lincoln, & Cabral, 2008; Heptinstall, Sethna, & Taylor, 2004; Ruf, Schauer, & Elbert, 2010; Schauer et al., 2003; Thabet, Abed, & Vostanis, 2004), a “building block” effect was demonstrated. The greater the exposure to traumatic stressors, the more intense the trauma-related symptoms up to levels when every survivor presents with PTSD; there is no ultimate resilience to repeated exposure to traumatic stress, each event adds-up to the destruction of the mental health (Neuner et al., 2004).

In line with the changes to the DSM-5 diagnostic criteria for PTSD (APA, 2013), the exposure to traumatic events can lead to the development of trauma-related guilt and shame (Kubany et al., 1996). High levels of guilt and shame may hinder the processing of what has happened, contributing to the development of mental disorders such as PTSD, also in children and adolescents (Lee, Scragg, & Turner, 2001; Muris & Meesters, 2014). However, shame and guilt have not been previously investigated in a population of refugee minors.

In agreement with Hagenaars, Fisch, and van Minnen (2011), the findings of the present study indicate a relationship between multiple trauma exposure and pathological shame with a higher number of traumatic events being associated with a higher level of shame in refugee minors. In addition, significant correlations between the intensity*duration*frequency of shame episodes and PTSD symptom severity could be found. Although Hagenaars, Fisch, and van Minnen (2011) state that the level of shame varies with the number of traumatic experiences, but is independent from PTSD symptom severity, the results of the present study indicate that the PTSD symptom severity is a significant predictor of pathological feelings of shame, accounting for 45% of the variance in the outcome variable.

In accordance with Henning and Frueh (1997), the PTSD sum score and its subscales, intrusion, avoidance, and hyperarousal, were significantly associated with the intensity*duration*frequency of shame episodes experienced by the refugee minors.

Prevalence rates of trauma-related guilt reported by refugee minors were 19% for global guilt, 26% for the affective component, and 22% for the cognitive component of guilt. This finding is consistent with a study conducted by Fletcher (2003). In response to a single traumatic event such as a dramatic accident or a natural disaster, about one-third of the children displayed feelings of guilt. In contrast, more than half of the children reported feelings of guilt when traumatic events were recurring as is the case with physical or sexual abuse. Because in most cases refugee minors reported recurring and long-lasting traumatic experiences in addition to openly expressed fears of potential negative consequences for the asylum procedure, rates in the present study are rather underestimated than overestimated.

According to Kubany and Watson's (2003) model of guilt, global guilt experienced in response to a traumatic event or series of events is a function of its affective and cognitive components. Consequently, an individual experiences trauma-related guilt when he or she is emotionally harmed (distress) by the traumatic event(s), and additionally takes responsibility (total guilt cognitions) for the circumstances or the results. Kubany and Watson (2003) state that guilt cognitions exhibit a strong effect on the severity of guilt-related distress, in contrast, a small effect is hypothesized from distress to guilt cognitions.

In the present study, the number of traumatic events was strongly related to the affective (distress) but not the cognitive component of guilt or global guilt. If a correlational pattern did exist, it would be weak, explaining <10% of the variance. Larger samples would be needed to model the interrelations between cumulative exposure to traumatic stressors, PTSD symptoms, and guilt components. The present results may encourage such further investigations examining how guilt components interact with each other to influence posttraumatic psychopathology.

There is substantial evidence that cognitions play an important role in the maintenance and chronicity of posttraumatic distress (Ehlers & Clark, 2000; Kubany et al., 1996; Kubany & Watson, 2003; Steil & Ehlers, 2000). When examining guilt cognitions in refugee adolescents, a correlation with PTSD symptom severity was found. This association can be explained by the fact that many trauma survivors exaggerate or distort the importance of their roles in the traumatic event or series of events (Kubany & Manke, 1995). Kubany and Manke (1995) identified four kinds of false beliefs held by trauma survivors: beliefs about (1) outcome foreseeability and preventability, (2) insufficient justification, (3) responsibility for causing trauma-related outcomes, and (4) wrongdoing. In retrospect, a victim may be convinced that he or she dismissed or overlooked clues and that the trauma-related outcomes were foreseeable and, therefore, preventable. Faulty conclusions intensify the level of distress, which in turn is significantly associated with PTSD symptom severity. As expected, a positive relationship between global guilt and PTSD symptom severity was found in the present study proving evidence for the maladaptivity of guilt. Similarly, a positive association between PTSD symptom severity and the intensity*duration*frequency of shame episodes indicates the maladaptivity of shame. However, the authors do not expect the self-conscious emotions to be maladaptive *per se* and, therefore, suggest further investigation of their components as well as their interactions. It could well be possible that reasoning about personal failure and guilt may support an effort of the individual to try and be in control when having to face adversities next time. In the same manner, feelings of shame could serve a purpose: they could be a consequence of the biological threat of social devaluation, rejection, or exclusion. As emotional action-eliciting dispositions, they are important to prevent expulsion or being socially alienated (Eisenberger, Lieberman, & Williams, 2003; Iffland, Sansen, Catani, & Neuner, 2014; MacDonald & Leary, 2005).

Moreover, adolescents who showed high levels of psychological distress, and hence received a PTSD diagnosis, reported significantly higher levels of the affective component of guilt than their peers who displayed subclinical symptoms. This most widely supports the findings of Nader et al. (1990) who examined the feelings of guilt in children after exposure to a school shooting. Correspondingly, a quarter of the children who received a PTSD diagnosis demonstrated feelings of guilt, whereas children without a diagnosis experienced no guilt.

Failure to detect the hypothesized associations with total guilt cognitions and global guilt may be due to a lack of power. However, by examining the components of trauma-related guilt simultaneously, the present study also draws attention to the relationship between guilt-related distress and posttraumatic psychopathology. Specifically, results indicate that higher levels of guilt-related distress may be related to more severe PTSD symptoms and PTSD diagnosis. Beck et al. (2011) explored regression models predicting PTSD symptoms among women who had experienced intimate partner violence. Within their study, distress assessed by means of the TRGI was the only guilt-related component found to consistently predict PTSD symptoms. Thus, further research is needed to empirically evaluate the multidimensional conceptual model proposed by Kubany and Watson (2003).

Furthermore, an examination of the items of the TRGI distress scale provides an indication that the findings may have been influenced by limitations related to conceptualization and measurement of the distress component. Some TRGI items do not explicitly state guilt-related distress as generated by guilt cognitions (e.g., “What happened caused me emotional pain”). Therefore, it is unclear whether the items assess distress related to guilt or more broadly measure emotional and physical distress related to the trauma memory. Although it is generally considered that guilt consists of an affective component (e.g., Foa, Steketee, & Rothbaum, 1989; Kubany & Watson, 2003; Resick, Nishith, Weaver, Astin, & Feuer, 2002), further discussion about the definition and conceptualization of guilt-related distress is much needed.

A Mann–Whitney test revealed significant differences between unaccompanied and accompanied minors regarding the level of total guilt cognitions, guilt-related distress, and pathological feelings of shame. Because of the higher number of traumatic events, both trauma-related guilt and pathological shame need to be addressed in most of the unaccompanied minors.

Furthermore, all components of guilt were found to be positively related to pathological shame. A stepwise regression displayed global guilt and its affective component as significant predictors of pathological feelings of shame. Thus, it needs to be further examined whether a correction of the distorted cognitive beliefs in Cognitive Therapy for Trauma-Related Guilt (Kubany & Manke, 1995) directly affects shame or primarily reduces the level of distress, thereby modulating the level of shame. However, as stated above, guilt and shame are self-conscious emotions that can lead to self-control: being evaluated while being the evaluator. One's failure to stay in control during the event, then leads to the fear of social consequences, that is, social exclusion. Visible display of guilt and shame in facial expression and behavior could serve the function of social inclusion when having caused harm or done wrong (Allen & Badcock, 2003). Evolutionary biologists explain that guilt-based submission “survivor guilt” can be caused by worrying about harming others. In this manner, survivor guilt promotes group cohesion, inhibits anti-social competition, and leads individuals to engage in altruistic behavior (O'Connor, Berry, Weiss, Schweitzer, & Sevier, 2000). Thinking along this line, it turns out that social inclusion as an urgent biological need plays an existentially important role for unaccompanied refugee children, who stand alone without their parents and community.

In the present study, we examined the complex relationship between trauma exposure and self-conscious emotions, first accounting for differences regarding the social contexts of UMR and AMR. However, the current study presents with some limitations. We did not investigate females, as the vast majority of the unaccompanied minors have been reported to be male (Bundesamt für Migration und Flüchtlinge, 2009). The limited sample size is due to the lack of a German central register of refugee minors which renders accessibility difficult. The sample was, of course, limited to individuals who agreed to participate in the study. It is possible that those with very high levels of symptoms were more motivated or, on the opposite, were more fearful to accept our invitation to participate in the study. Moreover, social desirability may bias answers in any such study, we therefore specifically emphasized the strict confidentiality prior to the interviews. In addition, feelings of shame tend to be hidden from others and, therefore, may not have been openly expressed by all refugee minors.

## Conclusions

Young refugees who experienced war events and displacement show elevated rates of psychopathology, especially PTSD. Shame and guilt have been recognized as important self-conscious emotions in individuals who have experienced trauma. The present study focused on the association between trauma-related guilt, shame, and PTSD in a community-based sample of UMR and AMR residing in southern Germany.

UMR reported a higher number of traumatic events, including war and armed conflict, and more often received a PTSD diagnosis than their accompanied peers. The number of traumatic events was found to be significantly associated with the severity of PTSD symptoms experienced by the adolescents. Thus, we conclude that the repeated exposure to traumatic stressors causes cumulative harm to the mental health of refugee minors.

In the present study, a positive relationship between multiple trauma exposure and pathological shame was found. Moreover, the number of traumatic events experienced was strongly related to guilt-related distress. However, no significant association with the cognitive component or global guilt could be found.

By examining the components of trauma-related guilt simultaneously, results specifically draw attention to the relationship between the affective component of guilt and posttraumatic psychopathology. Therefore, further investigation of the guilt components as well as their interactions is suggested.

As expected, global guilt and its components, as well as pathological shame, were shown to be highly correlated with PTSD symptom severity indicating the maladaptive effects of trauma-related guilt and shame. This study illustrated a significantly higher rate of traumatic events and higher levels of PTSD symptom severity in UMR. Thus, it is little surprise that unaccompanied minors also reported significantly higher levels of total guilt cognitions, guilt-related distress, and pathological feelings of shame in comparison to their accompanied peers.

In conclusion, early recognition and treatment of both trauma-related guilt and pathological shame are essential when working with adolescent refugees in order to prevent or reduce trauma-related distress and psychopathology.

## Supplementary Material

The relationship between trauma, shame, and guilt: findings from a community-based study of refugee minors in Germany

The relationship between trauma, shame, and guilt: findings from a community-based study of refugee minors in Germany
