# Supplementary material for: The relationship between trauma, shame, and guilt: findings from a community-based study of refugee minors in Germany
Source: Eur J Psychotraumatol. 2015 Jun 22;6:10.3402/ejpt.v6.25863. doi: 10.3402/ejpt.v6.25863 (PMC4478074; doi:10.3402/ejpt.v6.25863)
Supplement: The relationship between trauma, shame, and guilt: findings from a community-based study of refugee minors in Germany [file EJPT-6-25863-s001.pdf]

The relationship between trauma, shame and guilt: findings from a community-based study of refugee minors in Germany

Sabrina J. Stotz, Thomas Elbert, Veronika Müller & Maggie Schauer

Zusammenfassung

Theoretischer Hintergrund: Der vorliegende Beitrag verfolgt das Ziel, Scham- und Schulderleben minderjähriger Flüchtlinge in Bezug zur Anzahl der erlebten traumatischen Stressoren darzustellen. Es wurde vermutet, dass nicht nur die Symptomschwere der Posttraumatischen Belastungsstörung (PTBS), sondern auch traumaassoziiertes Scham- und Schulderleben mit zunehmender Erfahrung traumatischer Stressoren ansteigen würden. Ferner wurden unbegleitete mit begleiteten minderjährigen Flüchtlingen verglichen, wodurch ein Zusammenhang zwischen diesem sozialen Faktor und der gezeigten Psychopathologie erstmals genauer spezifiziert werden konnte.
Methoden: Im Rahmen der vorliegenden Studie wurden ausführliche psychodiagnostische Interviews mit 18 unbegleiteten minderjährigen Flüchtlingen (UMF) und 14 begleiteten minderjährigen Flüchtlingen (BMF) durchgeführt. Alle Probanden waren männlich und befanden sich zum Untersuchungszeitpunkt im Alter zwischen 11 und 20 Jahren. Die diagnostische Abklärung einer PTBS sowie der Symptomschwere erfolgte anhand des UCLA PTDS Index für DSM-IV. Zur Messung von traumaassoziierter Schuld wurde das Trauma-related Guilt Inventory (TRGI) eingesetzt. Die Schwere, Häufigkeit und Dauer erlebter Schamepisoden wurden mit Hilfe des Shame Variability Questionnaire (SVQ) erfasst.
Resultate: Das Erleben von Scham und Schuld sowie die Symptome der PTBS sind positiv mit der Anzahl erlebter Traumata assoziiert. Für das Scham- und Schulderleben der minderjährigen Flüchtlinge zeigten sich entsprechende Zusammenhänge mit der Symptomschwere der PTBS.
Schlussfolgerungen: Unbegleitete minderjährige Flüchtlinge berichteten von einer größeren Anzahl traumatischer Erlebnisse und erhielten häufiger die Diagnose einer PTBS als begleitete minderjährige Flüchtlinge. In der vorliegenden Studie konnte gezeigt werden, dass die kumulativen Effekte (Baustein-Effekt) von wiederholten traumatischen Erlebnissen einen Risikofaktor für die psychische Gesundheit von Flüchtlingskindern und -jugendlichen darstellen. Ferner geht das wiederholte Erleben traumatischer Stressoren mit traumassoziierter Scham und Schuld einher, welche den Leidensdruck der Betroffenen verstärken, in weitreichenden psychosozialen Einschränkungen resultieren und therapeutische Anforderungen erhöhen.

Citation: European Journal of Psychotraumatology 2015, 6: 25863 - http://dx.doi.org/10.3402/ejpt.v6.25863
