# Supplementary material for: The relationship between trauma, shame, and guilt: findings from a community-based study of refugee minors in Germany
Source: Eur J Psychotraumatol. 2015 Jun 22;6:10.3402/ejpt.v6.25863. doi: 10.3402/ejpt.v6.25863 (PMC4478074; doi:10.3402/ejpt.v6.25863)
Supplement: The relationship between trauma, shame, and guilt: findings from a community-based study of refugee minors in Germany [file EJPT-6-25863-s002.pdf]

Başlık: Travma, utanç ve suçluluk arasındaki ilişki: Güney Almanya'daki göçmen azınlık üzerine topluluk temelli çalışmadan bulgular

Sabrina Jessica Stotz

Özet
Arkaplan: Özellikle sık sık çoklu stresöre maruz kalan göçmenlerdeki travmatik stres ile utanç ve suçluluk gibi kendilik bilinci duygular arasındaki ilişki tamamıyla araştırılmak için bekleyen bir konu.
Amaç: Bu çalışmanın amacı göçmen azınlıklardaki utanç ve suçluluk duygularını araştırmak ve daha büyük kümülatif travmatik stresörlere maruz kalmanın ne ölçüde sadece daha ciddi TSSB semptomlarıyla değil utancın ve suçluluğun daha yüksek seviyeleriyle de sonuçlanabileceğini değerlendirmektir.
Yöntem: Almanya'daki sığınma evi/akıl hastanesi aradıkları dönemde hepsinin 18 yaş altı olduğu 32 erkek göçmen azınlık çalışmaya katılmayı kabul etti. Değerlendirme sırasında, yaşları 11 ile 20 arasında değişmekteydi. 18 göçmen, ev sahibi ülkeye akrabaları olmaksızın ulaşmıştı ("yalnız azınlıklar"). Yapılandırılmış tanı görüşmelerinde, bir TSSB tanısı UCLA TSSB Indeksi kullanılarak oluşturuldu. Travma sonrası suçluluk Travma alakalı Suçluluk Envanteri (Trauma-related Guilt Inventory; TRGI) ile değerlendirildi ve Utanç Değişkenliği Anketi(Shame Variability Questionnaire; SVQ) utanç dönemlerinin yoğunluğu, süresi ve sıklığını kaydetmek için kullanıldı.
Sonuçlar: Suçluluk ve utanç duyguları travma semptomları gibi kişinin deneyimlediği travmatik olay tipinin sayısı ile ilişkiliydi. Travma sonrası suçluluk ve utanç duygularının ikisi de TSSB semptomunun ciddiyeti ile ilişkiliydi.
Tartışma: Bulgular çoklu travmatik olaya maruz kalma gibi kümülatif stresin utanç ve suçluluktan dolayı daha çok acı çekme ve işlevsel zayıflamayı içeren akıl sağlığı için risk faktörleri oluşturduğunu gösterdi.

Anahtar Kelimeler: Suçluluk; Travmaya maruz kalma; Travma Sonrası Stres Bozukluğu; Göçmen azınlıklar

Name of translator: Seray Akça

Citation: European Journal of Psychotraumatology 2015, **6**: 25863 - http://dx.doi.org/10.3402/ejpt.v6.25863
